# Impact of respiratory cycle during mechanical ventilation on beat-to-beat right ventricle stroke volume estimation by pulmonary artery pulse wave analysis

**DOI:** 10.1186/s40635-024-00618-7

**Published:** 2024-04-09

**Authors:** Arnoldo Santos, M. Ignacio Monge-García, João Batista Borges, Jaime Retamal, Gerardo Tusman, Anders Larsson, Fernando Suarez-Sipmann

**Affiliations:** 1 Intensive Care Medicine Department. Hospital, Universitario Fundación Jiménez Díaz. IIS-FJD, Madrid, Spain; 2 CIBER de Enfermedades Respiratorias CIBERES ISCIII, Madrid, Spain; 3 Universidad Alfonso X El Sabio, Madrid, Spain; 4 Unidad de Cuidados Críticos, Hospital Universitario SAS de Jerez, Jerez de La Frontera, Spain; 5 First Faculty of Medicine, Institute of Physiology, Charles University, Prague, Czechia; 6 Departamento de Medicina Intensiva, Facultad de Medicina, Pontificia Universidad Católica de Chile, Santiago, Chile; 7 Department of Anesthesia, Hospital Privado de Comunidad, Mar del Plata, Argentina; 8 Department of Surgical Sciences, Uppsala University, Uppsala, Sweden; 9 Department of Critical Care, Hospital Universitario de la Princesa, Madrid, Spain

**Keywords:** Stroke volume, Right ventricle, Mechanical ventilation, Pulse wave analysis, Heart lung interactions, Fluid responsiveness

## Abstract

**Background:**

The same principle behind pulse wave analysis can be applied on the pulmonary artery (PA) pressure waveform to estimate right ventricle stroke volume (RVSV). However, the PA pressure waveform might be influenced by the direct transmission of the intrathoracic pressure changes throughout the respiratory cycle caused by mechanical ventilation (MV), potentially impacting the reliability of PA pulse wave analysis (PA_PWA_). We assessed a new method that minimizes the direct effect of the MV on continuous PA pressure measurements and enhances the reliability of PA_PWA_ in tracking beat-to-beat RVSV.

**Methods:**

Continuous PA pressure and flow were simultaneously measured for 2–3 min in 5 pigs using a high-fidelity micro-tip catheter and a transonic flow sensor around the PA trunk, both pre and post an experimental ARDS model. RVSV was estimated by PA_PWA_ indexes such as pulse pressure (SV_PP_), systolic area (SV_SystAUC_) and standard deviation (SV_SD_) beat-to-beat from both corrected and non-corrected PA signals. The reference RVSV was derived from the PA flow signal (SVref).

**Results:**

The reliability of PA_PWA_ in tracking RVSV on a beat-to-beat basis was enhanced after accounting for the direct impact of intrathoracic pressure changes induced by MV throughout the respiratory cycle. This was evidenced by an increase in the correlation between SVref and RVSV estimated by PA_PWA_ under healthy conditions: rho between SVref and non-corrected SV_SD_ – 0.111 (0.342), corrected SV_SD_ 0.876 (0.130), non-corrected SV_SystAUC_ 0.543 (0.141) and corrected SV_SystAUC_ 0.923 (0.050). Following ARDS, correlations were SVref and non-corrected SV_SD_ – 0.033 (0.262), corrected SV_SD_ 0.839 (0.077), non-corrected SV_SystAUC_ 0.483 (0.114) and corrected SV_SystAUC_ 0.928 (0.026). Correction also led to reduced limits of agreement between SVref and SV_SD_ and SVSyst_AUC_ in the two evaluated conditions.

**Conclusions:**

In our experimental model, we confirmed that correcting for mechanical ventilation induced changes during the respiratory cycle improves the performance of PA_PWA_ for beat-to-beat estimation of RVSV compared to uncorrected measurements. This was demonstrated by a better correlation and agreement between the actual SV and the obtained from PA_PWA._

**Supplementary Information:**

The online version contains supplementary material available at 10.1186/s40635-024-00618-7.

## Background

Estimation of left ventricle stroke volume (SV) through systemic arterial pulse wave analysis [1] is widely accepted in clinical practice [2]. The physiological principles used to estimate SV from arterial pulse wave analysis in the systemic circulation can also be applied to the pulmonary artery (PA) [3] to estimate the right ventricle (RV) SV (RVSV). Recent studies have underscored the role of RV and pulmonary circulation during hemodynamic instability situations [4, 5]. Consequently, there is a growing interest in real-time pulmonary hemodynamics monitoring at the bedside. In this context, beat-to-beat assessment of RVSV and their changes throughout the respiratory cycle provide complementary information that may help improve patient management. For example, this information could be applied for predicting RV fluid responsiveness and assessing the impact of mechanical ventilation (MV) on RV function.

Given the PA is located within the thoracic cage, RVSV estimation by pulse wave analysis may be influenced by respiratory-driven changes in the intrathoracic pressure. It is well-known that the intravascular pressure of cardiovascular structures within the thorax is sensitive to intrathoracic pressure [6]. While this is taken into account when evaluating measures, such as cardiac preload, the impact of the direct transmission of continuous intrathoracic pressure fluctuations on PA pressure waveform, especially those induced by MV, still remains poorly explored.

There are two reasons for which this becomes particularly pertinent in the context of PA pulse wave analysis (PA_PWA_) to estimate RVSV: (1) the interplay between stroke volume and arterial properties may be better represented in transmural than in intravascular pressure and (2) if direct transmission of respiratory changes alter PA pressure morphology, consecutive measurements could yield inaccurate conclusions when estimating RVSV variations during the respiratory cycle.

In this study, we hypothesized that MV-induced changes throughout the respiratory cycle could compromise the accuracy of PA_PWA_ in deriving beat-to-beat RVSV from PA pressure. To validate this hypothesis, we proposed a method for correcting the influence of MV on continuous PA pressure readings throughout the respiratory cycle and assessed its performance on estimating RVSV by PA_PWA_. This involved a retrospective analysis of data from mechanically ventilated pigs monitored with high fidelity PA pressure and flow sensors, both under healthy conditions and after the development of acute respiratory distress syndrome (ARDS).

## Methods

This study was conducted at the Hedenstierna laboratory, Uppsala University, Sweden and approved by the Institutional Animal Care and Use Committee. Five pigs (*sus scrofa domesticus,* Norwegian–Yorkshire breed) were studied. These animals are part of previous published studies [7, 8] performed in 2013 and were chosen if the same PEEP was applied before and after ARDS development, there was no exposure to vasoactive drugs during the study period, an absence of spontaneous respiratory efforts during data collection, and the ability to ensure simultaneous and continuous recordings of both airway and PA pressure and flow.

### Anesthesia, instrumentation and ARDS model

Instrumentation and model have been described elsewhere [7, 8] as well as within the online supplement. In summary, anesthetised and mechanically ventilated animals were tracheotomised and subjected to a small lateral thoracotomy to place a 20–24 mm ultrasonic flow probe (COnfidence Flowprobes, PAU series, Transonic, Ithaca, NY, USA) around the main PA and a micro-tip pressure transducer (Mikro-Tip, SPR-340, Millar, Houston, TX, USA) directly into the PA. A 4-lm central catheter and a PA catheter (Edwards, Irvine, CA, USA) were inserted through the right internal jugular vein. Animals were continuously monitored with electrocardiogram, femoral artery pressure for arterial gas samples, volumetric capnography and airway flow and pressure (NICO®, Philips, Wallingford, CT, USA).

After instrumentation, animals were subjected to a lung volume history homogenization manoeuvre [7, 8]. Baseline measurements were obtained 15 min thereafter with MV set with PEEP 8 cmH_2_O, tidal volume 6–8 ml/kg, I:E 1:2, FIO_2_ 1 and respiratory rate adjusted to keep an end-tidal CO_2_ around 45 mmHg in volume-controlled ventilation.

ARDS was created by performing saline lung lavages followed by 2 h of injurious ventilation. Once the model was established, ventilation parameters were changed to baseline and a new set of measurements was obtained after stabilization for 1 h.

A continuous 2–3 min stable signal period was selected at baseline and after ARDS. We tried to avoid extrasystoles and other undesired variability sources during selection of these recordings. These conditions were chosen for two reasons: (1) to evaluate the correction method in conditions where respiratory mechanics were different, as this factor could influence the transmission of airway pressure to the vascular structures and (2) to assess the impact of changes in pulmonary vascular mechanics on the performance of the proposed method, as the first is known to be affected and could alter PA pressure waveform in ARDS [7].

### Correction of the MV effect on continuous measurement of PA pressure

During MV, airway pressure fluctuates throughout the respiratory cycles leading to alterations in intrathoracic pressure that are directly transmitted to the PA pressure. We applied a method to correct this effect by tracking the PA diastolic pressure throughout the respiratory cycle to estimate the intrathoracic pressure changes and subtract them from the PA pressure signal. The rationale is grounded on the notion that late into the diastole, the influence of external pressure on a vessel would be more pronounced, since the impact of cardiac contraction would have largely dissipated.

**Fig. 1:**
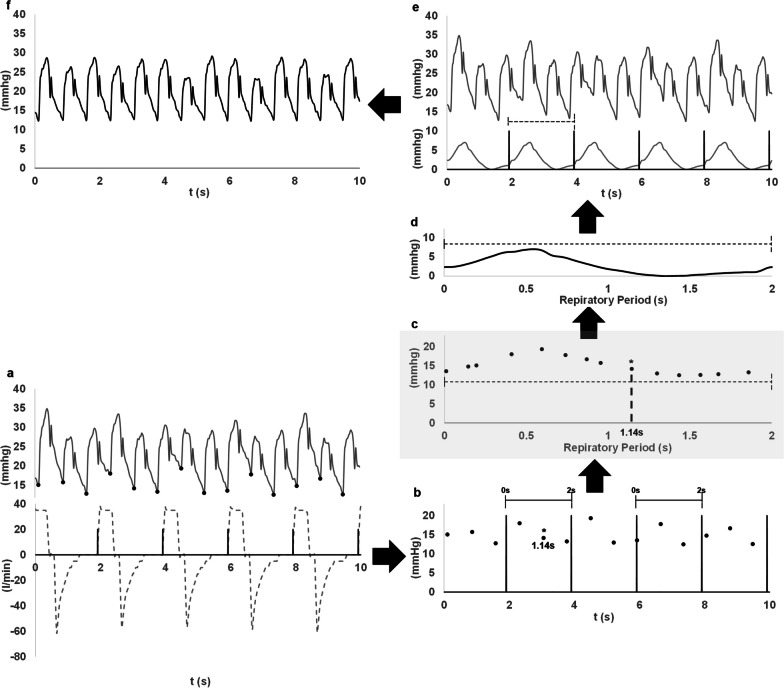
Algorithm to correct the effect of breathing on pulmonary artery pressure signal. See the description in the manuscript. As noted, the respiratory and pulmonary artery pressure signals should be recorded simultaneously. In brief, the following steps are shown: **a** minimum pressure (black points) at the start of the pulmonary artery pressure (continuous grey line) cycle and the start of inhalation (black lines) in the respiratory flow (dashed grey lines) cycle are located. **b** Time of minimum pulmonary artery pressure points in relation with the respiratory cycle period are set (dashed lines demark the respiratory period). One of the minimum pulmonary artery pressure points is marked with a star and its relative position according to the respiratory period is shown. **c** Minimum pulmonary artery pressure points are reordered according to their relative position in the respiratory cycle. This step is highlighted in grey due to its relevance for the method. The new location of the point marked with a star in **b** is now shown as an example of this step. **d** Function of the minimum pressure vs it relative time in the respiratory period is created. The function is scaled to its minimum value. Dashed line demark the respiratory period. **e** Function is used to create the continuous signal (bottom continuous grey line) along the analysed period and subtracted from the original pulmonary artery pressure signal (top continuous grey line). The dashed line demarks the respiratory period. **f** Corrected pulmonary artery pressure results from the above mentioned subtraction

To discern the direct impact of MV on PA waveform, we performed the following steps (Fig. 1):

1. We identified the minimum pressure at the foot of each PA pressure cycle using an iterative algorithm and the electrocardiogram as fiduciary signal to separate the cardiac cycles (Fig. 1a).
2. Simultaneously, each respiratory cycle was identified using the airway flow signal and setting a threshold of 5lpm for defining the beginning of the inspiration (Fig. 1a).
3. The time scale was then adjusted to start at each respiratory cycle initiation (Fig. 1b).
4. The beginning of each cardiac cycle was marked according to the new time scale, i.e. according to the relative position of the minimum pressure in the respiratory period (Fig. 1b).
5. Subsequently, cardiac beats were rearranged based on the timing of the minimum pressure value in relation to the respiratory cycle (Fig. 1c). This procedure implies using the respiratory period in the x axis instead of the time scale.
6. Using these reordered values, we constructed a function that represents the minimum pressure points across the respiratory period. An interpolation technique using a smoothing splines algorithm was applied to estimate missing values ensuring a degree of signal smoothness without significantly altering the primary curve shape (Fig. 1d).
7. Signal was then scaled by subtracting its lowest value and applying this adjusted function throughout the evaluation timeframe at intervals demarcated by the respiratory cycle (Fig. 1d, e).
8. In the final step, we subtracted this signal from the recorded PA pressure waveform (Fig. 1e), yielding the corrected PA pressure signal (Fig. 1f). An example of the non-corrected and corrected PA pressure signals in relation to the airway pressure is shown in Additional file 1: eFigure1.

Signal acquisition and processing is further described in the online supplement.

### Beat-to-beat measurement of RVSV and PAPWA variables for its estimation

On a beat-to-beat basis, the following variables were calculated on PA flow or pressure signals:

- **Stroke volume reference (SVref):** Area under the flow-time curve during systole.
- **PA pulse pressure:** The difference between PA systolic and foot pressure.
- **PA pressure systolic area:** Area under the PA pressure curve, starting at pressure foot and concluding at the dicrotic notch.
- **PA pressure standard deviation:** Pressure SD throughout each pressure cycle.

A detailed description of the methods applied to calculate these variables is provided in the online supplement (Additional file 1: eFigures 2 and 3). Although such methods have been originally developed for systemic arterial pulse wave analysis, the physiological principle, where they grounded should theoretically stand for the PA_PWA_ as well. We assessed the performance of SV obtained from PA pulse pressure (SV_PP_), systolic area (PA_SystAUC_) and standard deviation (SV_SD_) derived from both the non-corrected and corrected pressure signals in tracking RVSV.

### Evaluation of the impact of breathing on beat-to-beat tracking of RVSV using PA_PWA_

When using pulse pressure, standard deviation and systolic pressure area for SV estimation by pulse wave analysis, a calibration factor is required [1]. This factor depends mainly on vascular properties, and it is assumed to be constant along the respiratory cycle [9]. In our study, this calibration factor was derived from the ensemble average of 10 PA pressure and flow consecutive cycles obtained during an expiratory pause at each studied situation. The SV obtained from the resulting mean cycle of flow was divided by the evaluated variable (pulse pressure, standard deviation, and systolic area) calculated from the mean cycle of pressure. The calibration factor was utilized for SV derivation from PA_PWA_ variables on a beat-to-beat basis and was applied for both non-corrected and corrected signals.

The SV variation during a respiratory cycle (SVV) [10] was used to quantify the known effect of heart–lung interactions on SV:

SVV = (SVmax − SVmin)/[SVmax + SVmin)/2] [11]where SVmax and SVmin are the maximum and minimum SV during a respiratory cycle. The SVV was calculated for PA_PWA_ SV and for the SVref, computing it for each respiratory cycle over the studied period. The median SVV over the studied period was used as representative of each animal and condition.

For assessing the impact of our correction on the respiratory and cardiac components of the PA pressure signal, a frequency domain analysis was applied. This entailed the generation of an amplitude spectrum on PA pressures segments of 2 min, applying a fast Fourier transform algorithm with a 16,384 size, a Hamming window and 75% overlapping. Noise was defined as a value < 1% of the maximum amplitude of the corresponding spectrum. Respiratory and cardiac components were identified according to the respiratory and heart rates during each study period. The sum of the area under the curve of the 1st–4th harmonics of the respiratory rate was used to calculate the respiratory component, and of the 1st–12th harmonics of the heart rate for the cardiac component.

### Statistical analysis

Normalcy was assessed using the Shapiro–Wilk test. Data were expressed as mean (SD) if it followed a normal distribution or otherwise as median [25th–75th interquartile range]. As beat-to-beat SVref and PA_PWA_ variables did not follow a normal distribution during periods of analysis, median was chosen as representative of each animal and condition. Similarly, the median absolute deviation (MAD) [12] and the MAD divided by median (MAD/MED) were calculated as measures of variability for each animal and condition. Paired *t* test and Wilcoxon signed-rank test (for non-normally distributed data) were used for comparing measurements in baseline versus ARDS and for comparing non-corrected versus corrected PA_PWA_ variables. Two-way repeated measures ANOVA was applied to evaluate the effect of ventilation correction and lung condition (Baseline and ARDS), on PA_PWA_ derived variables. Pearson’s R (for normal distributed data) or Spearman’s Rho (for non-normal distributed data) were calculated to evaluate the correlation between SVref and PA_PWA_ SV and the SVV calculated from them. Cross-correlation between SVref and PA_PWA_ SV was used to test if the effect of MV on measured PA pressure caused a phase shift between flow and pressure signal. A phase shift was defined as an increase in correlation in any lag between ± 1 and ± 4. McNemar’s test was used to test if the proportion of animals in which a phase shift was observed was different with or without the applied correction. To compare lung condition and the effect of correction, a representative value was obtained for each evaluated variable from each animal and condition during the analysis period (for example, Rho between SVref and SV_PP_). Then, mean (SD) or median according [25th–75th interquartile range] from the 5 animals at each lung condition was used to perform the corresponding comparisons. It was assumed that the effect of correction was specific for each animal, so the correction function was calculated for each individual and situation. However, to characterize the impact of the proposed correction method on the evaluated data set, a mixed effect linear regression of all the measures was used using SV obtained from PA_PWA_ as the dependent variable, SVref, correction and lung condition as fixed effects, and animal as random effect. A Bland–Altman analysis corrected for repeated measurements [13] of all the comparison pairs was performed at each lung condition and the percentage error [14] was calculated to represent the change in agreement caused by the introduction of the correcting method. Significance was considered at *p* < 0.05. Bonferroni correction was applied to account for multiple comparisons. Statistical analysis was performed with Microsoft excel 2013 (Microsoft Corporation, Redmond, WA, USA) and Stata v15 (StataCorp, College Station, TX, USA).

## Results

Five pigs (31.8 ± 6.0 kg) were studied. A total of 1328 (266 ± 65 per animal) heart beats were analysed at baseline and 1595 (319 ± 61 per animal) after ARDS. The induction of ARDS resulted in significant worsening of respiratory mechanics, gas exchange and pulmonary hemodynamics (Table 1). Tidal overdistension was also evident according to the increased stress index [15].

**Table 1:** Respiratory and pulmonary hemodynamic variables during the studied situations

Variable	Baseline	ARDS	*p*
V_T_ (ml/kg)	8.48 [7.04–8.59]	8.52 [5.85–8.76]	0.500
RR (bpm)	32 (2)	33 (3)	0.570
PEEP (cmH_2_O)	8.1 (0.1)	8.1 (0.2)	0.711
PPlat cmH_2_O)	15.5 [12.9–15.7]	27.7 [26.9–29.9]	**0.043**
MAwP (cmH_2_O)	10.9 (0.6)	14.4 (0.7)	**< 0.001**
DP (cmH_2_O)	7.3 [4.9–7.5]	19.4 [18.8–22.2]	**0.043**
Cdyn (ml/cmH_2_O)	39.5 (7.4)	11.8 (2.3)	**0.002**
SI	1.04 [0.95–1.06]	1.16 [1.16–1.16]	**0.043**
PaO_2_ (mmHg)	541 (49)	196 (108)	**0.005**
PaCO_2_ (mmHg)	42 (6)	59 (12)	**0.019**
SaO_2_ (%)	98.8 (0.8)	95.8 (2.82)	0.069
pH	7.43 (0.06)	7.27 (0.06)	**0.027**
Shunt	0.09 (0.05)	0.25 (0.10)	0.060
CO (l/min)	3.19 [2.91–3.20]	2.71 [2.38–3.82]	0.500
HR (bpm)	96 (17)	107 (21)	0.257
PAPm (mmHg)	20 (2)	31 (6)	**0.007**
PVR (dyn.s.cm^−5^)	172 (65)	577 (156)	**0.005**
PA_Comp_ (ml/mmHg)	3.37 [3.10–5.65]	1.71 [1.35–2.04]	**0.043**

P values lower than 0.050 were higlighted in bold

Respiratory and hemodynamic variables at baseline and after acute respiratory distress syndrome (ARDS) was developed

V_t_ tidal volume, RR respiratory rate, SaO_2_ arterial haemoglobin O_2_ saturation, PEEP positive end expiratory pressure, PPlat plateau pressure, MAwP mean airway pressure, DP driving pressure (DP = PPlat-PEEP), Cdyn respiratory system dynamic compliance, SI stress index, CO cardiac output, HR heart rate, PAPm mean pulmonary artery pressure, PVR pulmonary vascular resistance, PA_Comp_ pulmonary artery compliance (stroke volume divided by pulmonary artery pulse pressure)

The effects of correction and lung conditions on the PA_PWA_-derived RVSV are summarized in Table 2 and in the online supplement. The application of the correction method slightly but significantly modified the SV_SD_ and SV_SystAUC_ both at baseline and ARDS. The SV_PP_ decreased after ARDS induction with and without the correction of the effect of MV. The correction procedure resulted in a reduced variability in SV_PP_ and SV_SystAUC_ throughout the evaluation period, as indicated by the MAD and MAD/MED. Results for non-calibrated PA_PWA_ variables are shown in the Additional file 1: eTable 2.

**Table 2:** Evaluation of the effect of correction on right ventricular stroke volume derived from pulse wave analysis

Correction	Cond	SVref	SV_PP_		SV_SD_		SV_SystAUC_	
			NC	Cor	NC	Cor	NC	Cor
Stroke volume	Baseline	35.8 (2.9)	40.9 (6.6)	40.7 (7.0)	36.0 (2.9)	37.1 (2.8)^c^	39.4 (4.5)	38.1 (4.4)^c^
(ml)	ARDS	31.2 (7.1)	32.1 (7.7)^a^	31.6 (7.4)^a^	29.8 (7.2)	31.3 (7.2)^c^	32.0 (8.0)	33.4 (8.4)^c^
MAD (ml)	Baseline	2.2 (0.6)	4.4 (1.4)	1.9 (0.5)^c^	1.6 (0.5)	1.8 (0.3)	1.9 (0.6)	1.2 (0.5)^c^
	ARDS	2.1 (0.4)	3.0 (0.5)	1.4 (0.4)^c^	1.3 (0.6)	1.6 (0.5)	1.7 (0.4)	1.2 (0.4)^c^
MAD/med	Baseline	0.06 (0.02)	0.11 (0.03)	0.05 (0.01)^c^	0.05 (0.01)	0.05 (0.01)	0.05 (0.02)	0.03 (0.01)^c^
	ARDS	0.07 (0.02)	0.09 (0.01)	0.05 (0.00)^c^	0.04 (0.01)	0.05 (0.01)	0.05 (0.01)	0.04 (0.01)^c^
Rho	Baseline	N.A	0.863 (0.70)	0.882 (0.092)	− 0.111 (0.342)	0.876 (0.130)^c^	0.543 (0.141)	0.923 (0.050)^c^
	ARDS	N.A	0.752 (0.080)	0.848 (0.049)	− 0.033 (0.262)	0.839 (0.077)^c^	0.483 (0.114)	0.928 (0.026)^c^
SVV (%)	Baseline	20 (6)	31 (6)	18 (4)^c^	17 (9)	20 (6)	14 (4)	12 (6)
	ARDS	23 (5)	37 (7)	23 (6)^c^	17 (3)	25 (7)	15 (3)	17 (6)

Stroke volume, MAD (median absolute difference) and MAD/MED (MAD divided by median) were obtained from all the beat-by-beat measurements during the studied period at each animal and condition. Rho represents the correlation between evaluated variable estimated from PA PWA and SVref evaluated beat-by-beat. SVV is the stroke volume variation and it was calculated at each respiratory cycle during the studied period. The representative value of each variable is the mean (SD) (according to distribution of data) for the 5 animals in the evaluated condition

^a^*p* < 0.05 for lung condition ^c^*p* < 0.05 for the correction

A detailed description of the statistics results is provided in the online supplement

P values lower than 0.050 were indicated in bold

**Fig. 2:**
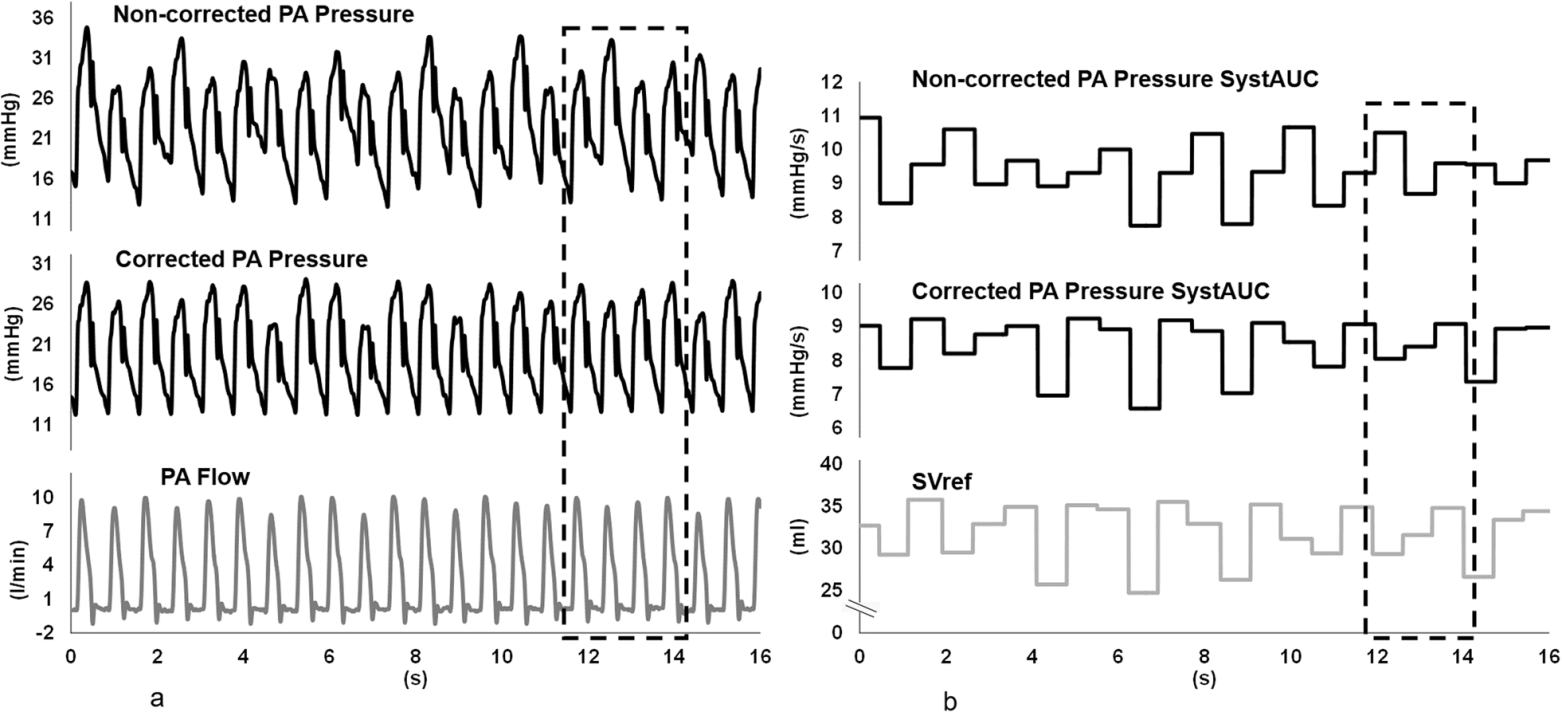
Case example of the beat-by-beat variability of the studied signals. **a** Example of the beat-by-beat variability of Non-corrected and Corrected PA Pressure and flow (PA Flow). Note how the synchronized changes on pressure and flow cycles are more evident when PA pressure signal is corrected (dashed rectangle). **b** Example of beat-by-beat measurement of reference stroke volume from the PA flow signal (SVref) and systolic area from non-corrected and corrected PA pressure. As shown in the dashed rectangle, the changes in SVref and Corrected PA Pressure SystAUC are in phase while the SVref and Non-corrected PA Pressure SystAUC are decoupled

**Fig. 3:**
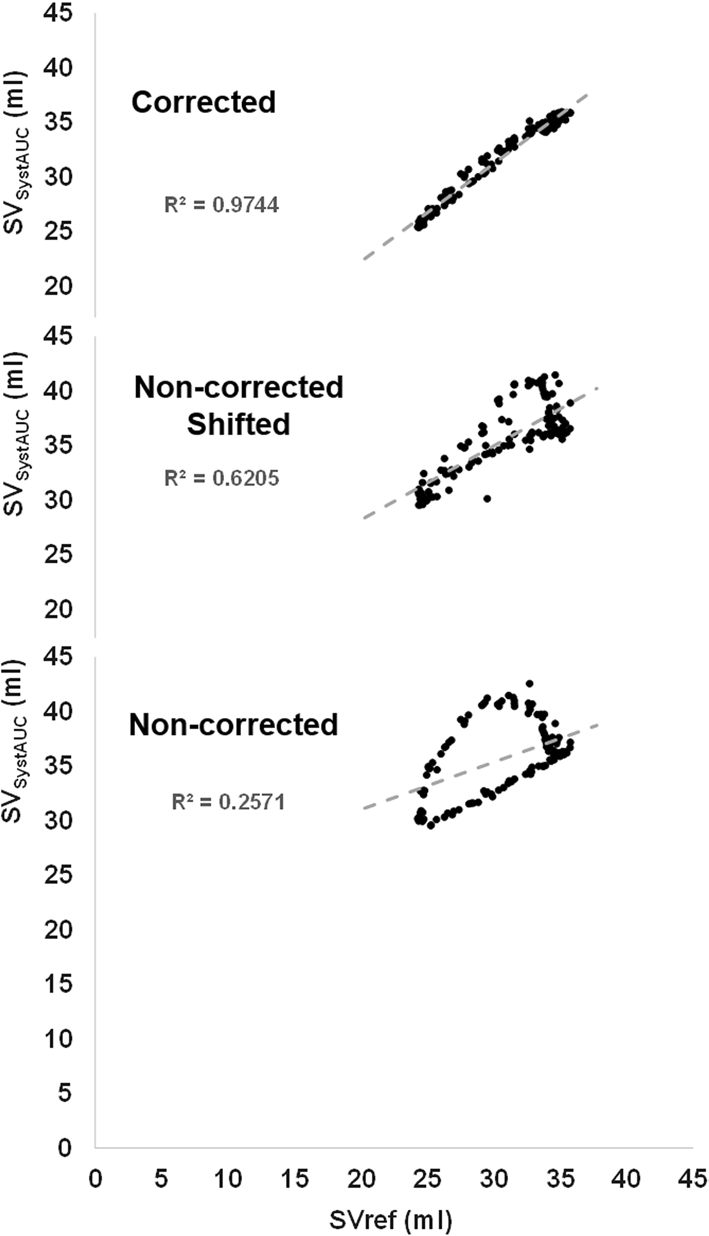
Partial shift of PA pressure and effect of correction. A case example of the scatter plot between SV estimated from systolic PA pressure area SV SystAUC and the reference stroke volume (Svref) is shown. The decoupling between the SystAUC SV and the SVref results on a nonlinear relationship and low correlation when the PA pressure is non-corrected (lower panel). This effect is partially corrected when the signals are shifted (middle panel). And totally corrected when the correction is applied (upper panel). To improve the quality of the image, we used a lower number of matched pairs to construct the plots that those analysed in this animal and condition (140 out of 266)

A case illustration highlighted a mismatch between PA pressure systolic area and the SVref when relying upon the non-corrected signal. This discrepancy was not observed in the corrected signal (Fig. 2). This was also corroborated in the scatter plot of SVref vs SV_SystAUC_ (Fig. 3). While a shift adjustment in the signals partially corrected this discrepancy, the proposed method removed it. The cross-correlation assessment also shows a phase shift in both SV_SD_ (in ARDS) and SV_SystAUC_ (both at baseline and ARDS), which was rectified post-correction (*p* = 0.025) (Additional file 1: eTable3). A similar pattern was observed for SV_PP_, where a shift in signals for non-corrected pressure was recorded in 60% of ARDS cases, and for SV_SD_ in 80% of baseline cases, although the correction did not lead to any statistical significance. Beat-to-beat correlation between SVref and SV_SD_ and SV_SystAUC_ was enhanced when MV effect was corrected. A similar trend was observed for SV_PP_ after correction, although it did not reach statistical significance (Table 2).

**Fig. 4:**
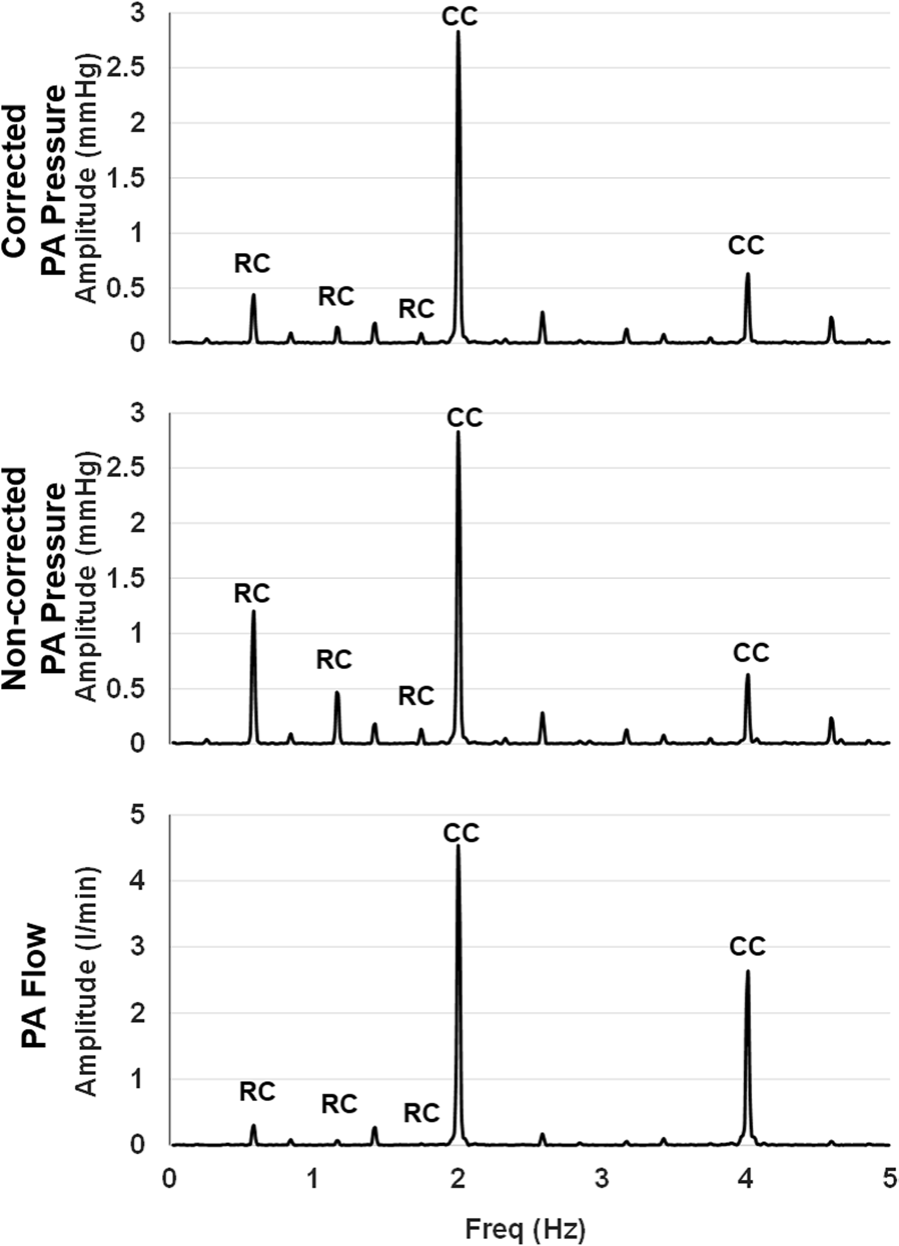
Example of the pulmonary artery pressure and flow spectrum. An example of the amplitude spectrum of pulmonary artery flow (lower panel, PA Flow), non-corrected PA pressure (middle panel) and corrected PA pressure (upper panel). Respiratory and cardiac component (RC and CC respectively) were recognized by the natural respiratory and heart rate during the studied condition. Note how correction decreases the respiratory component while keeps intact the cardiac component

**Fig. 5:**
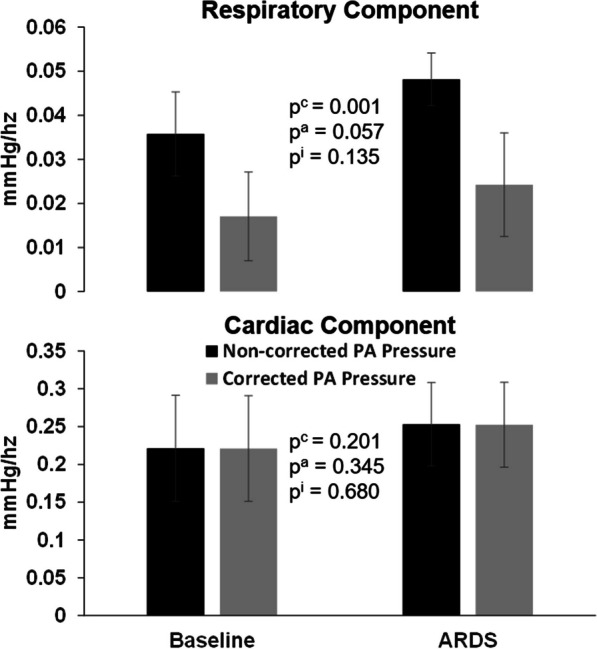
Respiratory and cardiac component of PA pressure and flow spectrum. Comparison of the effect of correction on respiratory and cardiac component of the amplitude spectrum in baseline and ARDS. Correction decreases the respiratory component while keeps intact the cardiac component. ^c^*p* < 0.05 for the correction. ^a^*p* < 0.05 for lung condition. ^i^*p* < 0.05 for the interaction correction lung condition

In the frequency domain analysis, the correction noticeably reduced the respiratory component without affecting the cardiac component in both baseline and ARDS conditions, as shown in Figs. 4 and 5.

**Fig. 6:**
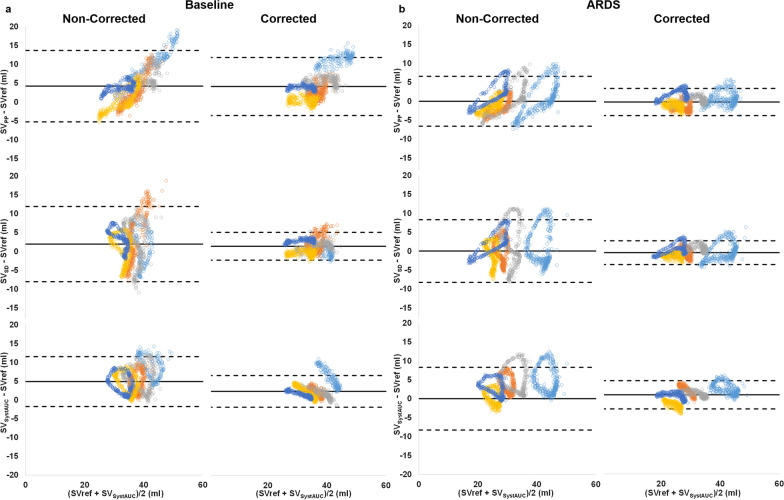
Bland–Altman plots of SV obtained from corrected and non-corrected PAPWA in baseline (**a**) and ARDS (**b**). Limits of agreement were adjusted for repeated measures. Each colour represents an animal. Top: SV obtained from PA Pulse pressure (SV_PP_). Middle: SV obtained from PA pressure standard deviation (SV_SD_). Bottom: SV obtained from PA systolic area (SV_SystAUC_)

Correction reduced the limits of agreement between SV obtained from PA_PWA_ and SVref at baseline and ARDS (Table 3, Fig. 6). A similar effect was observed for the percentage error (Table 3). A significant effect (*p* < 0.005) of correction was found in the mixed effects linear regression model for SV_SD_ and SV_SystAUC_. Plots of SV obtained from PA_PWA_ vs SVref are shown in Additional file 1: eFigure 4.

**Table 3:** Bland–Altman analysis of stroke volume obtained from PA_PWA_ analysis and reference

Baseline variable	Correction	Bias (ml)	LoA (ml)	Percentage error (%)
SV_PP_	Non-corrected	4.2	[− 5.2–13.7]	26
	Corrected	4.1	[− 3.6–11.8]	20
SV_SD_	Non-corrected	1.9	[− 8.0–11.9]	29
	Corrected	1.3	[− 2.4–5.0]	10
SV_SystAUC_	Non-corrected	4.9	[− 1.7–11.5]	19
	Corrected	2.3	[− 1.9–6.5]	11
ARDS				
SV_PP_	Non-corrected	0.0	[− 6.6–6.6]	21
	Corrected	− 0.1	[− 3.7–3.5]	11
SV_SD_	Non-corrected	0.0	[− 8.3–8.3]	27
	Corrected	− 0.5	[− 3.6–2.7]	10
SV_SystAUC_	Non-corrected	3.1	[− 3.4–9.6]	20
	Corrected	1.0	[− 2.8–4.7]	11

Stroke volume obtained from pulmonary artery pulse pressure (SV_PP_), standard deviation (SV_SD_) and systolic area (SV_SystAUC_). LoA limits of agreement

**Fig. 7:**
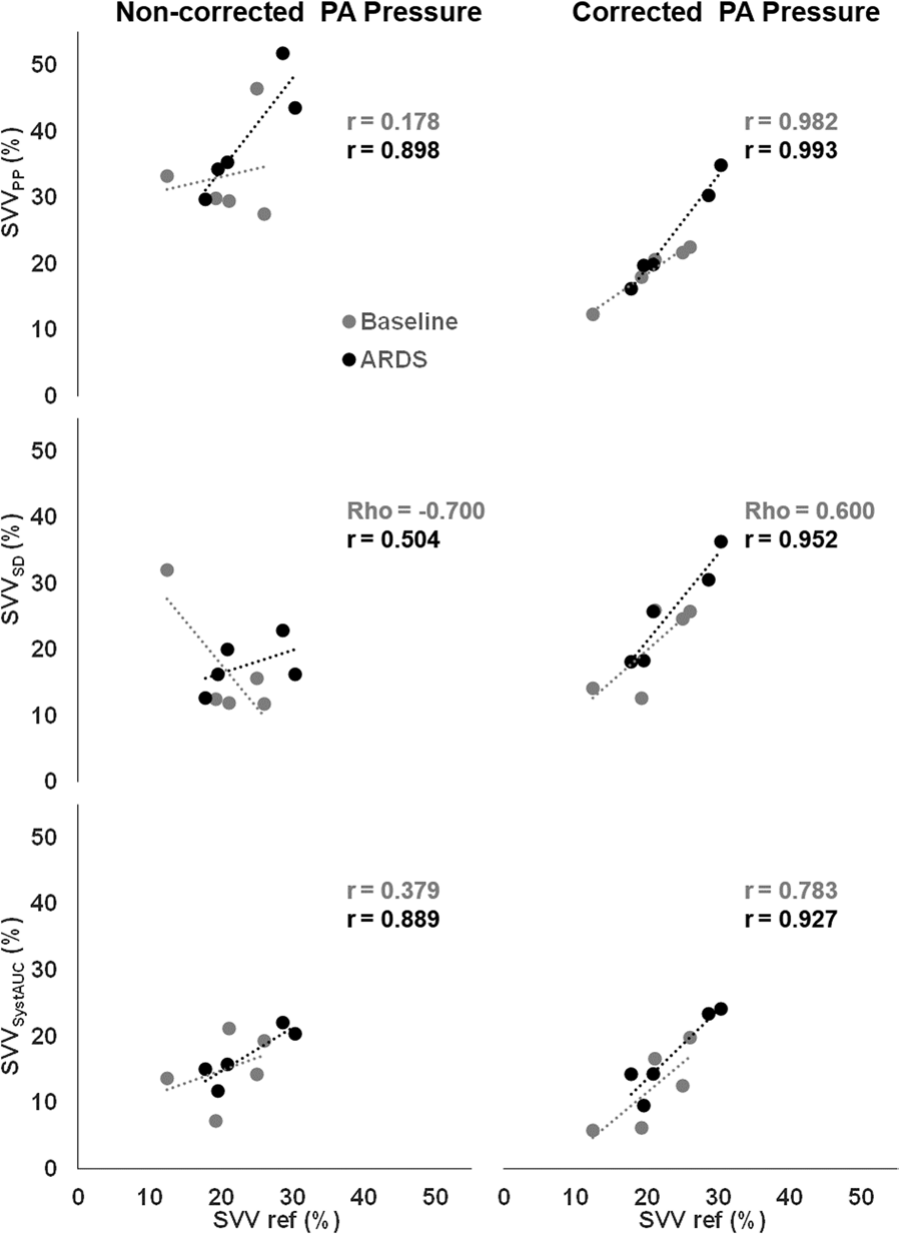
Effect of correction on Stroke volume variation. Correlation between the stroke volume variation calculated from the SVref (SVVref) and the SVV calculated from variables obtained from the Non-corrected (left) and Corrected (right) pulmonary artery pressure in Baseline (gray) and ARDS (black). SVV_SystAUC_, SVV_SD_ and SVV_PP_ are the SVV calculate from the systolic area, standard deviation and pulse pressure of the PA pressure respectively. Note how the correction improves the correlation for each variable and condition

After the application of the correction, the SVV obtained from SV_PP_ was significantly reduced (Table 2). A trend to increase the SVV calculated from SV_SD_ was also observed. The correlation between SVV obtained from SVref and SVV calculated from SV obtained from PA_PWA_ improved after correction (Fig. 7).

## Discussion

In our study, the influence of MV on intrathoracic pressure throughout the respiratory cycle and its subsequent effect on PA pressure measurements was analysed. Our results underscored that the MV-induced fluctuations in intrathoracic pressure have the potential to compromise the accuracy of PA_PWA_ in real-time tracking of RVSV on a beat-to-beat basis. These results are relevant for clinical practice as they serve as foundational knowledge for refining and optimizing PA_PWA_ algorithms. In particular, they could help to better describe the impact of ventilation on RVSV and pulmonary hemodynamics.

Our findings showed that the reliability of the three evaluated PA_PWA_ variables (pulse pressure, standard deviation and systolic area), substantially improved when specific corrections were applied. This improvement was manifest in the enhanced correlation and reduced limits of agreement (Table 3 and Fig. 6) observed between the SVref and the PA_PWA_ SV on a beat-to-beat basis. In addition, percentage error was decreased by correction. Although the observed percentage error lies below 30% [14], the precision method of our reference method for RVSV is higher than the thermodilution method, so a lower limit should be expected.

A partial phase shift might account for the discrepancies observed with non-corrected PA_PWA_ variables. This phase shift could stem from altered measured PA pressure caused by transmission of intrathoracic pressure fluctuations caused by MV. MV impacts RVSV by altering RV preload and afterload. In the absence of an external pressure, the pressure waveform primarily reflects the interplay between SV and pulmonary vascular properties. However, during MV an external pressure distorts the measured PA pressure. This external pressure is not necessarily in phase with SV changes driven by intrathoracic pressure fluctuations during MV. For example, a cardiac cycle during late inspiration faces higher external pressures yet might have a reduced preload due to diminished venous return, caused by higher intrathoracic pressures. This phenomenon was corroborated by the observed improvement in the correlation between PWA_PA_-derived variables and SV_ref_ after the phase shift correction and depicted in Figs. 2 and 3.

Another significant result of our study was that the correction of the estimated changes in the intrathoracic pressure minimized the respiratory component without altering the cardiac component from the PA pressure amplitude spectrum analysis (Figs. 4 and 5). This finding suggests that, while the effects of the direct transmission of the intrathoracic pressure were removed, the interaction between SV and vascular properties, which determines the PA waveforms, remained unaffected. Furthermore, even after correction, the physiological modulation of MV on RVSV by the known heart–lung interactions persisted, as demonstrated by the improved correlation between SVV obtained from the reference method and that obtained from the corrected PA_PWA_ variables (Fig. 7).

### Clinical application

The results of this work may have a translational value. First, continuous beat-to-beat tracking of RVSV is still lacking with the current monitoring tools. Traditional methods, such as thermodilution, only provide an average measure and, while they can estimate SV dividing the CO by heart rate, they cannot provide insights on a beat-to-beat scale. Newer monitoring devices, which incorporate algorithms to estimate SV and CO from PA_PWA_, also fall short as they calculate these values over extended time periods rather than on the precise scale of cardiac cycles; therefore, they do not allow evaluation of beat-by-beat variability nor the effect of breathing on SV. Previous experimental studies have shown the possibility of detecting short-term changes in SV with PA pulse contour but a low beat-by-beat correlation with the reference method [3]. While echocardiography and Doppler techniques do offer beat-scale estimations of RVSV, their intermittent nature precludes their feasibility for continuous monitoring.

Enhanced capability for estimating RVSV on a beat-to-beat basis may also offer advancements beyond the technical improvements in CO estimation. Another clinical challenge that this method could address is the independent assessment of fluid responsiveness in the left and right ventricles. The current paradigm categorizes patients broadly: patients are fluid responders only if both ventricles are preload-dependent, and non-responders if one of them shows no preload-dependency. Such generalized approach can be limiting. The ability to assess each ventricle’s fluid responsiveness state independently, has the potential to improve hemodynamic management algorithms. Interestingly, past attempts in this direction, such as those relying on PA pulse pressure variation, have failed to demonstrate any clinical usefulness [16]. However, the impact of correcting the effect of intrathoracic pressure fluctuations on PA pressure to assess RV fluid responsiveness needs to be evaluated in further studies.

Another application of this refined method of RVSV estimation holds the potential in understanding the interplay between ventilation and RV function. It is known that patients with ARDS are susceptible to RV failure, a condition that significantly impairs patient outcomes [17]. A major determinant of this phenomenon is the impact of mechanical ventilation on RV afterload, which could be evaluated, at least in part, by the continuous analysis of the RVSV modulation during the respiratory cycle.

We must point out the relevance of improving beat-to-beat calculation for the above-mentioned applications. Although methods such as averaging several cardiac cycles could increase the agreement of SV obtained from PA_PWA_, this could be at the expense of decreasing the measured effect of MV. As shown in Additional file 1: eTable 1 and eFigure 7, the limits of agreement and percentage error decrease by smoothing and are similar for both corrected and non-corrected data. In addition, a better correlation is observed in Additional file 1: eFigure 6. However, this is at the expense of a lower variability (Additional file 1: eFigures 5 and 6) and the amplitude of the fluctuation not only in PA_PWA_ variables but also in the SVref. As pointed out in the methods, the major source of SV variability in our data set for each analysis period (animal and condition) is mediated by physiological effects of MV. In this regard, for example, the calculus of SVV from averaged data would lead to erroneous lower values. According to this, while averaging could be enough to obtain representative values SV from PA_PWA_, our method outperforms this technique for identifying the changes of SV along a respiratory cycle. For the above proposed applications of beat-to-beat tracking, an accurate and precise estimation of the effect of MV on RVSV is necessary.

### Limitations

This is study is limited by its small sample size, reducing the statistical power in some evaluations. Furthermore, while we used a high-fidelity pressure sensor for our measurements, it remains to be demonstrated whether our findings can be generalized to data obtain from conventional PA catheters. Further research should explore this.

To correct the effect of MV throughout the respiratory cycle, an external signal for determining the start of respiratory cycle was needed. We used the airway flow signal for this purpose, however, other signals such as airway pressure and exhaled CO_2_ could also be used, and the ability of other methods to reliably separate the respiratory cycles (for example, using the PA pressure) could be explored.

In our settings, we were able to test the correction method in two different lung conditions: baseline (healthy lung) and ARDS. This allowed us to evaluate our method under healthy conditions and also in the presence of decreased lung compliance as well as increased RV afterload (Table 1). While a decreased lung compliance could decrease the transmission of changes in airway pressure caused by MV during the respiratory cycle, an increase in RV afterload could affect the physiologic effect of MV on RVSV. Our method was able (to contrast both conditions and) to catch the real change in stroke volume and also worked in the presence of altered lung mechanics. However, we were not able to test the effect of other relevant variables, such as PEEP or tidal volume. PEEP can also affect both lung compliance and pressure transmissibility and also, by affecting preload, the physiologic effect of MV on RVSV along the respiratory cycle. On the other hand, for a given preload status, tidal volume proportionally modifies the magnitude of SV variation caused by MV. Although, as the correction function is calculated for each condition and subject, it is expected that the proposed method remains reliable when changing the mentioned variable, this should be tested in further experiments. In the same line, we evaluate SVV within limited MV settings (PEEP 8 cmH_2_O and tidal volume around 8 ml/kg), and we were not able to test if our method would reliably sense the effects of these variables on the SVV.

## Conclusions

Under the described experimental conditions, we demonstrated that correcting for mechanical ventilation induced changes during the respiratory cycle improves the measurement performance of PA_PWA_ for beat-to-beat estimation of RVSV compared to an uncorrected measurement. This was demonstrated by a better correlation and agreement between the actual SV and the obtained from PA_PWA._ Such findings are potentially applicable at bedside and may help improve patient management.

### Supplementary Information

**Additional file 1:** Addtional methods and results.

## Data Availability

The data sets and used analysed during the current study are available from the corresponding author on reasonable request.

## Abbreviations

ARDS: Acute respiratory distress syndrome
MAD: Median absolute difference
MAD/MED: Median absolute difference divided by the median
MV: Mechanical ventilation
PA: Pulmonary artery
PA_PWA_: Pulse wave analysis of the pulmonary artery
PEEP: Positive end-expiratory pressure
RV: Right ventricle
RVSV: Right ventricle stroke volume
SD: Standard deviation
SV: Stroke volume
SVmax: Maximum stroke volume
SVmin: Minimum stroke volume
SV_PP_: Stroke volume obtained from the pulse pressure
SVref: Stroke volume obtained from the pulmonary artery flow
SV_SD_: Stroke volume obtained from the standard deviation
SV_SystAUC_: Stroke volume obtained from the systolic area
SVV: Stroke volume variation
