# Supplementary material for: Impact of respiratory cycle during mechanical ventilation on beat-to-beat right ventricle stroke volume estimation by pulmonary artery pulse wave analysis
Source: Intensive Care Med Exp. 2024 Apr 9;12:34. doi: 10.1186/s40635-024-00618-7 (PMC11004097; doi:10.1186/s40635-024-00618-7)
Supplement: Supplementary file 1 — Additional file 1: Addtional methods and results. [file 40635_2024_618_MOESM1_ESM.docx]

**Online Supplement to: Impact of Respiratory Cycle during Mechanical Ventilation on Beat-to-Beat Right Ventricle Stroke Volume Estimation by Pulmonary Artery Pulse Wave Analysis.**

**Authors**

Arnoldo Santos^1,2^, M. Ignacio Monge-García^3^, João Batista Borges^4^, Jaime Retamal^5^, Gerardo Tusman^6^, Anders Larsson^7^, Fernando Suarez-Sipmann^2,8^.

**Affiliations**

1. Intensive Care Medicine Department. Hospital Universitario Fundación Jiménez Díaz. IIS-FJD. Madrid, Spain

2. CIBER de enfermedades respiratorias CIBERES ISCIII. Madrid, Spain

3. Unidad de Cuidados Críticos, Hospital Universitario SAS de Jerez, Jerez de la Frontera, Spain

4. First Faculty of Medicine, Institute of Physiology, Charles University, Prague, Czechia.

5. Departamento de Medicina Intensiva, Facultad de Medicina, Pontificia Universidad Católica de Chile, Santiago, Chile.

6. Department of Anesthesia, Hospital Privado de Comunidad, Mar del Plata, Argentina.

7. Department of Surgical Sciences, Uppsala University, Uppsala, Sweden.

8. Department of Critical Care, Hospital Universitario de La Princesa, Madrid, Spain.

**Corresponding author**

Arnoldo Santos, Intensive Care Medicine Department. Hospital Universitario Fundación Jiménez Díaz. IIS-FJD. Madrid, Spain

Email: [asantosoviedo@yahoo.com](mailto:asantosoviedo@yahoo.com) Tel: +34915504800 ext 4671

ORCID: 0000-0003-4797-9677

**Extended methods**

*Anaesthesia, instrumentation and ARDS model*

After inducing anaesthesia, animals were tracheotomised and connected to mechanical ventilation (Servo-i. Maquet Critical Care, Solna, Sweden). Intravenous anaesthesia was maintained with ketamine, midazolam and fentanyl, adding rocuronium for muscle relaxation after adequate anaesthesia was ascertained.

After instrumentation, animals were subjected to a lung volume history homogenization manoeuvre in pressure controlled ventilation mode with positive end expiratory pressure (PEEP) 20 cmH_2_O, driving pressure 20 cmH_2_O, respiratory rate 20 bpm and I:E 1:1 for 30 seconds.

ARDS was created by performing saline (30 ml/kg) lung lavages until a PaO_2_/FIO_2_ < 200 mmHg was reached at PEEP 8 cmH_2_O and FIO_2_ 1.0. Animals were then subjected to two hours of injurious ventilation (PEEP 0 cmH_2_O and inspiratory driving pressure 35 cmH_2_O in pressure-controlled ventilation mode).

Intravenous boluses of normal saline (5ml/kg) were administered if mean systemic arterial pressure was < 60mmHg during the model creation.

*Signal acquisition and processing*

The flow probe was attached to a Perivascular Flow Module (TS420, Transonic, Ithaca, NY, USA) and the pressure probe to a PowerLab Data Acquisition System (PowerLab 16/35, Adinstruments, Dunedin, New Zealand). Both systems were connected to a MP-150 Biopac Data Acquisition System (MP-150WSW, Biopac Systems, Inc., Goleta, CA, USA) for simultaneous data acquisition together with electrocardiogram, airway pressure and flow and other physiological signals at 200 or 1000 Hz (later resampled to 200 Hz). Signals were processed using LabChart 8 software (Adinstruments, Dunedin, New Zealand). A Savitzky-Golay 2^nd^ order filter with an 11-points window was applied for smoothing. All signal analysis and calculations as well as the described methods for correcting PA pressure were performed using customized routines programmed in Excel (Microsoft Corporation, Redmond, WA, USA) and Matlab (Mathworks Inc, Natick MA, USA).

*Effect of the correction on the PA pressure signal according to respiratory cycle*

As shown in efigure 1, Airway pressure change during mechanical ventilation affects the PA pressure continuous waveform. In the example, the non-corrected signal increases during inspiration due in part to the direct transmission of airway pressure to the PA pressure. Such effect is removed by the proposed correction method while keeping the physiologic effect of mechanical ventilation on PA pressure modulation. Note that the correction signal is in phase with the airway pressure.


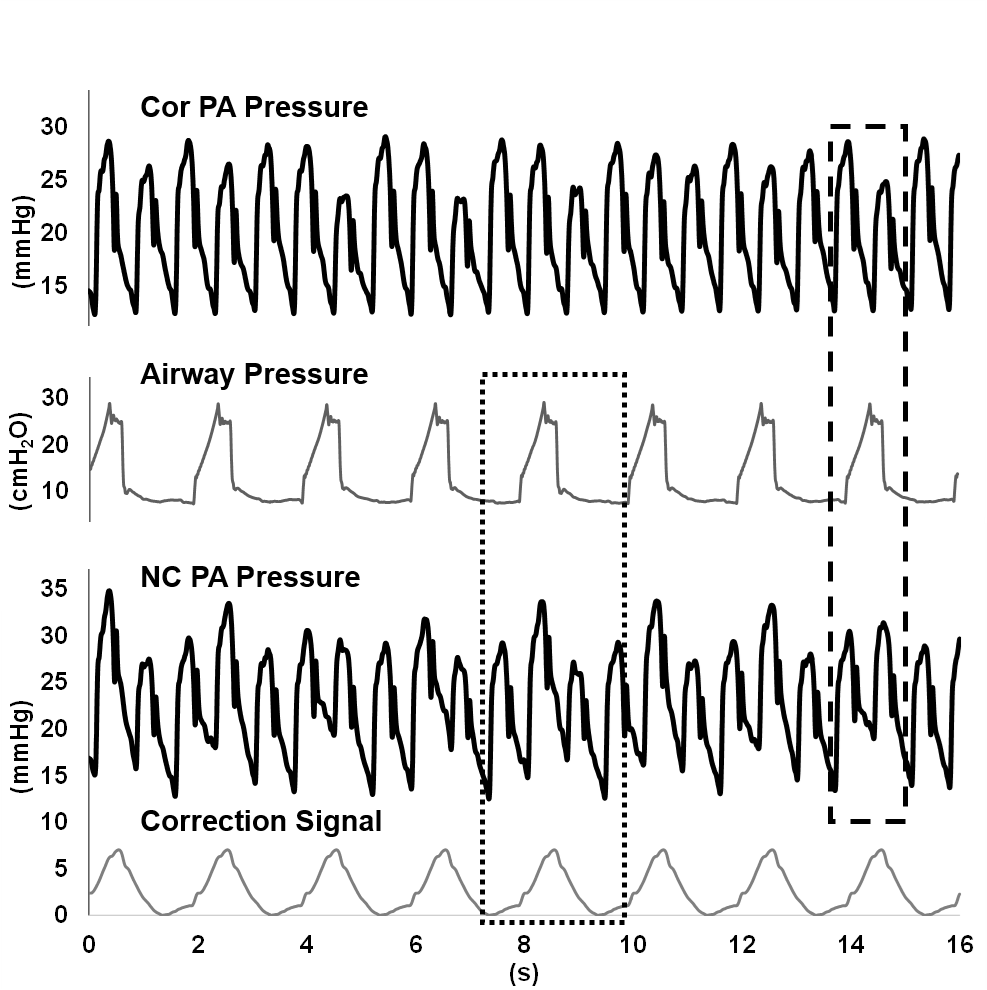


**efigure 1.** Example of the correction of pulmonary artery pressure signal. Corrected pulmonary artery pressure (Cor PA Pressure) is the result of subtracting the correction signal from the non-corrected PA pressure (NC PA Pressure). Correction signal is in phase with airway pressure (highlighted in dotted rectangle). Note how, despite of correction, physiological beat-to-beat variability remains in the Cor PA pressure. This variability is mainly due to the effect of breathing on right ventricle preload and afterload. Also, the effect of the correction on systolic variability is highlighted in the dashed rectangle.

*Beat-to-beat Measurement of Stroke Volume and PWA Variables for its Estimation*

Cardiac cycles were identified using the electrocardiogram as fiduciary signal. On the continuous PA flow signal, foot was determined as the first maximum of the second derivative of the flow signal. PA systolic flow was defined as the flow signal portion starting at the flow foot and ending just before first negative flow value (see efigure 2). Stroke volume reference was calculated as the area under systolic flow:

$\sum_{Qfoot}^{Qend ejection} Q\left( t \right)\Delta t$


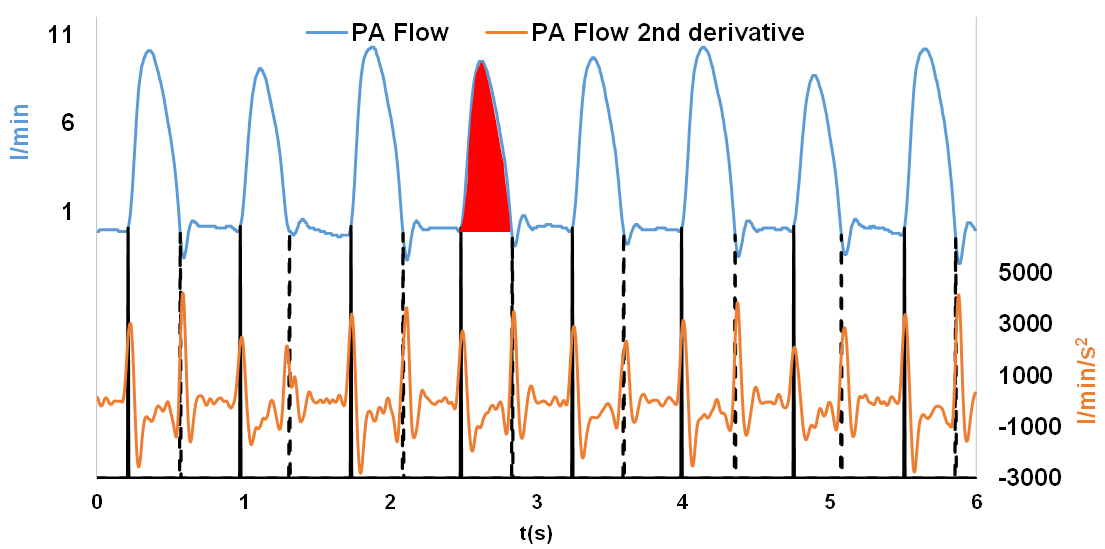


**efigure 2.** Simultaneous PA flow (blue continuous line) and its second derivative (orange continuous line). An example of the flow area (in red) used to calculate stroke volume is shown. Foots of the flow cycle (continuous black line) and flow cycle ends (dashed black lines) are shown.

PA pressure foot was defined as the first zero crossing (negative to positive) of the first derivative of pressure signal. PA systolic pressure was defined as the maximum value of the PA pressure cycle. PA Pulse pressure was calculated as systolic PA pressure minus PA pressure foot.

PA dicrotic notch was defined as the first zero crossing (from negative to positive) of the first derivative of pressure signal after the PA systolic pressure. PA systolic area was defined as the area under the PA waveform from PA foot to PA dicrotic notch and calculate as:

$$\sum_{Pfoot}^{P dicrotic} P\left( t \right)\Delta t$$

Average of the PA pressure cycle was calculated over the entire PA pressure cycle period (from foot to foot). Standard deviation was calculated as the average of the differences between all PA pressure points (n) and the average of the PA pressure cycle:

$$1/n\sum_{Pfoot}^{n} (P\left( t \right)-PAaverage)$$

An example of PA pressure signal with the points used to perform the above described calculations described above is shown in efigure3:


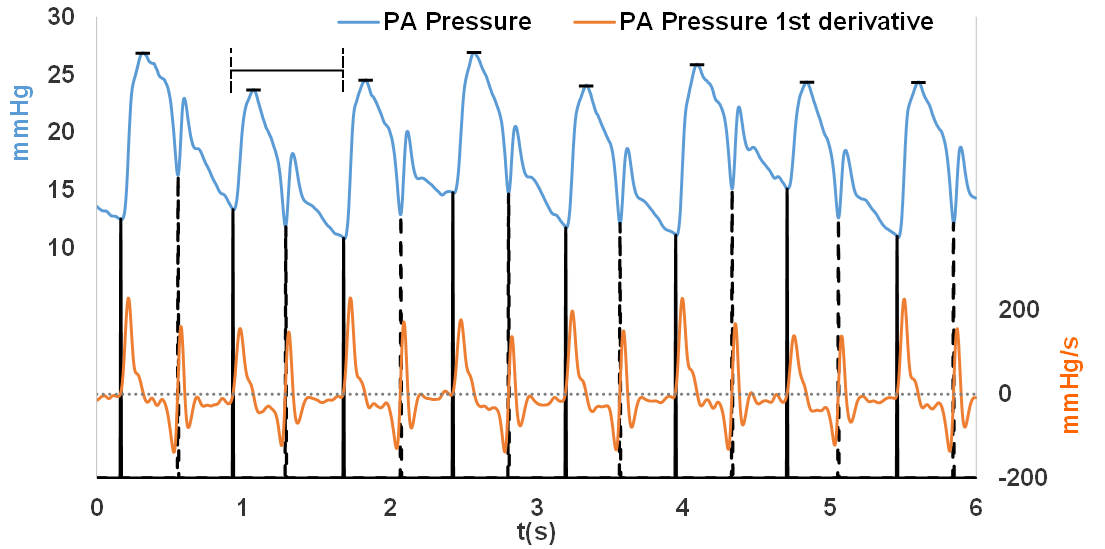


**efigure 3.** Simultaneous PA pressure (blue continuous line) and its first derivative (orange continuous line). Systolic area is calculated from PA pressure foot (continuous black line) to dicrotic notch (dashed black line). Pulse pressure was calculated as systolic (short black lines) minus PA pressure foot. The standard deviation was calculated from foot to foot. The zero level of the first derivative of pressure (used to find the pressure foot and the dicrotic notch) is shown in dotted grey line. The horizontal line above PA pressures signal indicates the duration of one pressure cycle.

**Extended results**

*Results of two way repeated measure ANOVA*

Following, we show the results of the repeated measures analysis evaluating the effect of correction of the effect of MV on PA pressure signal (correction) and ARDS (lung condition) in the 5 studied pigs (table 2 in the main text):

Stroke volume

PA_PWA_ Variable Factor F P

SV_PP_ Correction 13.21 0.022

Lung Condition 151.39 <0.001

SV_SD_ Correction 30.34 0.005

Lung Condition 7.02 0.057

SV_SystAUC_ Correction 42.96 0.002

Lung Condition 10.68 0.031

MAD

PA_PWA_ Variable Factor F P

SV_PP_ Correction 92.29 0.001

Lung Condition 0.00 0.987

SV_SD_ Correction 4.47 0.102

Lung Condition 0.28 0.623

SV_SystAUC_ Correction 98.37 0.001

Lung Condition 1.45 0.295

MAD/Med

PA_PWA_ Variable Factor F P

SV_PP_ Correction 136.13 <0.001

Lung Condition 0.63 0.472

SV_SD_ Correction 1.68 0.265

Lung Condition 0.01 0.939

SV_SystAUC_ Correction 100.78 0.001

Lung Condition 0.83 0.414

Rho

PA_PWA_ Variable Factor F P

SV_PP_ Correction 4.89 0.091

Lung Condition 2.69 0.180

SV_SD_ Correction 62.61 0.014

Lung Condition 0.07 0.808

SV_SystAUC_ Correction 97.05 0.001

Lung Condition 0.81 0.419

SVV

PA_PWA_ Variable Factor F P

SV_PP_ Correction 104.76 0.001

Lung Condition 1.86 0.245

SV_SD_ Correction 2.36 0.199

Lung Condition 0.53 0.508

SV_SystAUC_ Correction 0.00 0.968

Lung Condition 6.83 0.059

*Plots of the stroke volume obtained from PAPWA vs SVref*

Plots of the SV obtained from PA_PWA_ vs the SVref are shown in efigure 4.


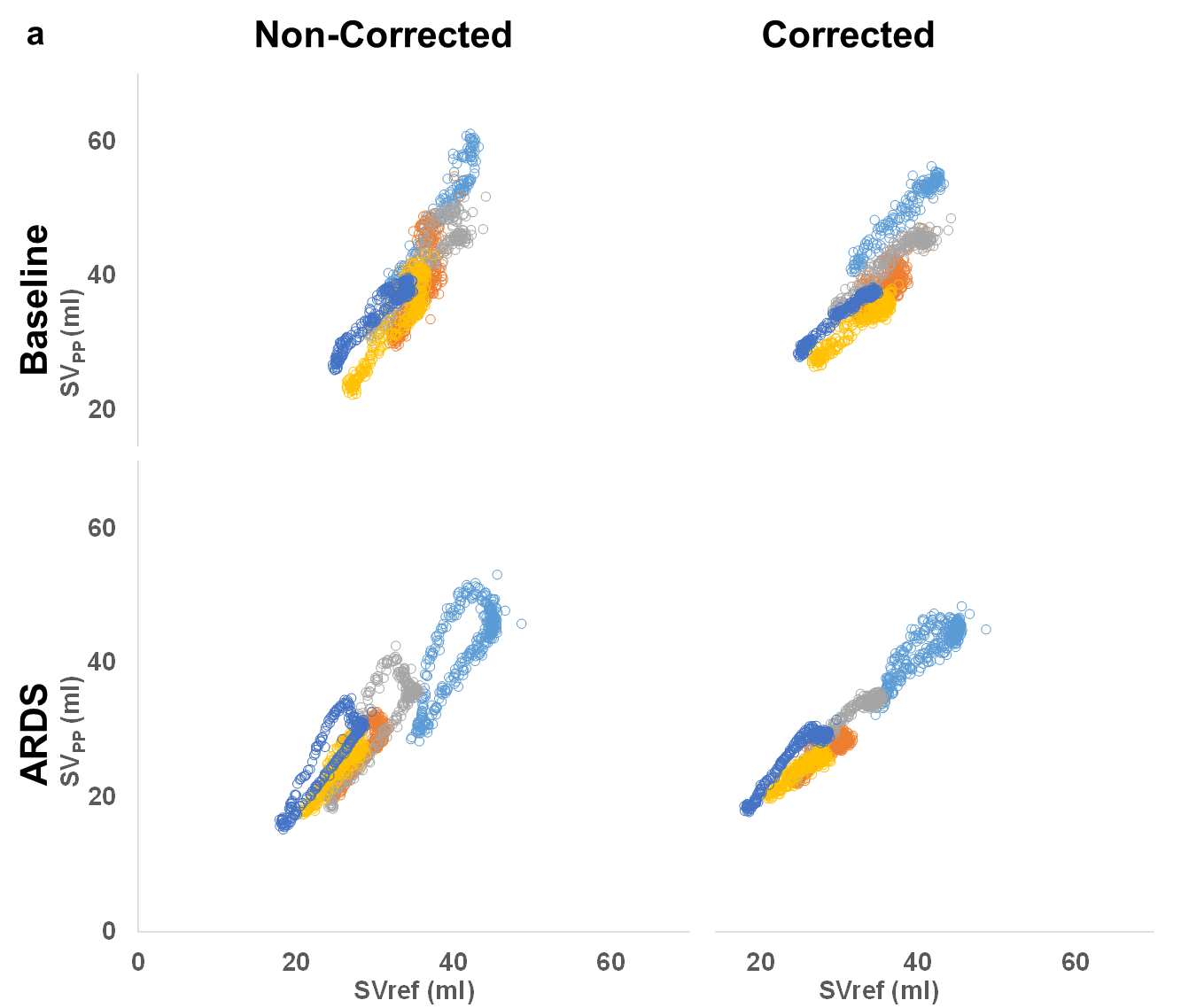


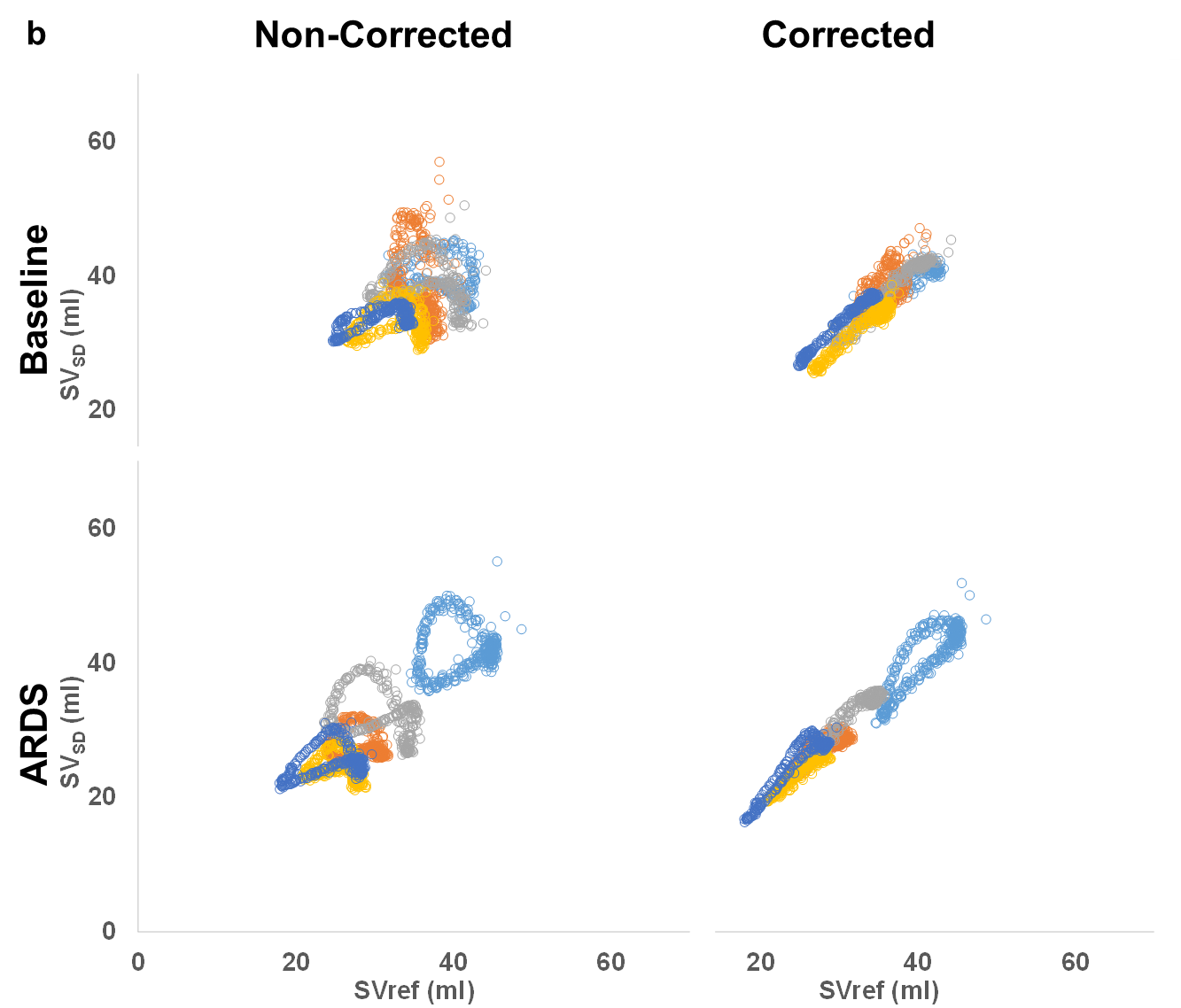


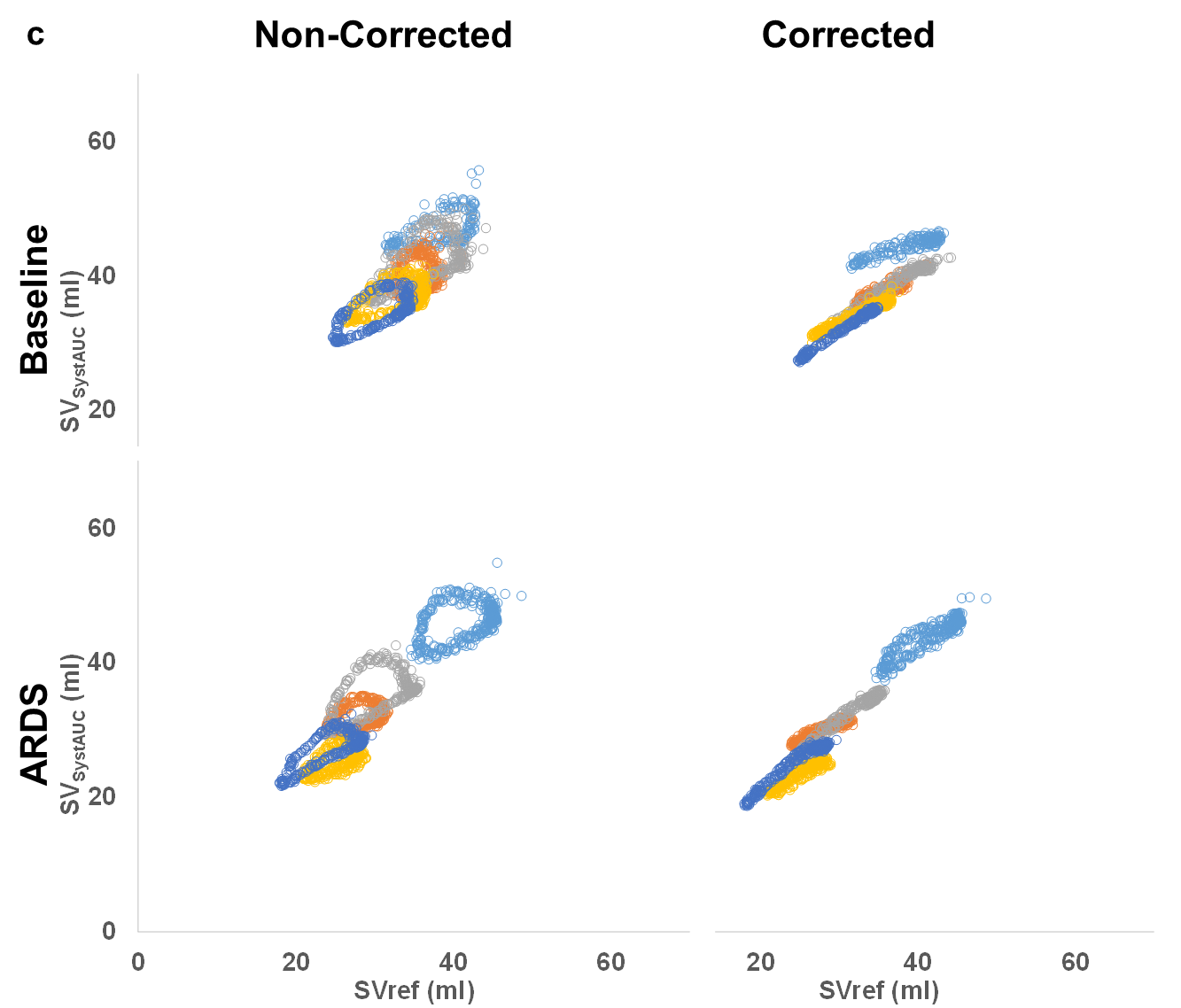


**eFigure 4.** PA_PWA_ SV vs SVref. Each colour represents an animal. **a.** SV obtained from PA Pulse pressure (SV_PP_). **b)** SV obtained from PA pressure standard deviation (SV_SD_). **c.** SV obtained from PA systolic area (SV_SystAUC_).

*Effect of smoothing /averaging*

As one of the known procedures to catch changes along time in continuous measurements decreasing variability is the application is by averaging we tested this complementary analysis in our data set. For such a purpose_,_ a 5 heartbeats moving average was applied on SV ref and SV obtained from corrected and non-corrected PA_PWA_. An example of the effect of this smoothing is shown on eFigure 6 and eFigure7.


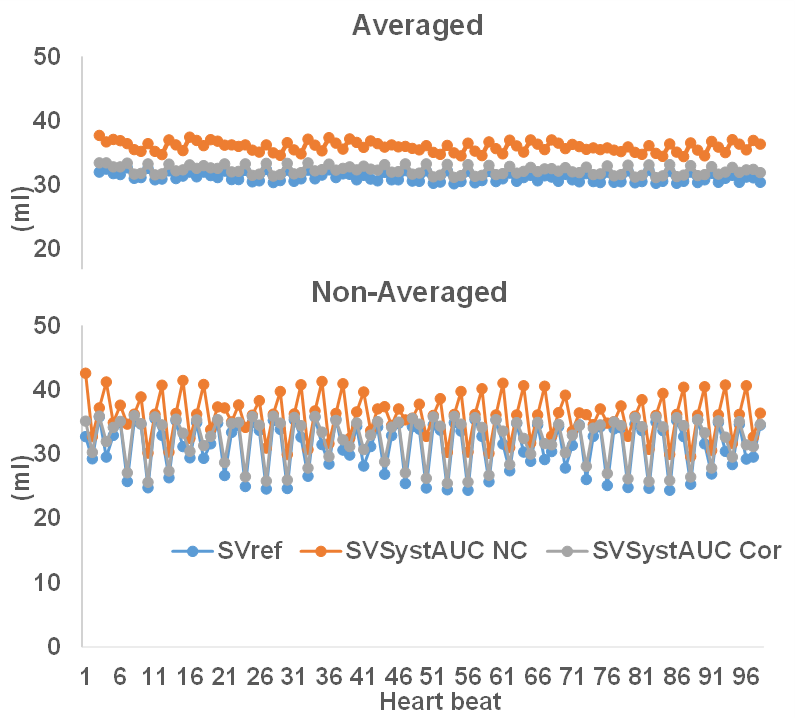


**eFigure 5.** Continuous heart beats of an example animal showing the effect of smoothing (above) comparing with non-smoothed (below) on the SVref (blue) and the stroke volume obtained from PA systolic area (SVSystAUC) non-correcting (NC, orange) and correcting (Cor, grey). Although smoothing decreased the difference between Svref and its estimation from PA_PWA_ the beat-to-beat variability at each signal is also decreased. Part of this variability if due to the physiologic variation on SV (see the SVref beat-to-beat variability).


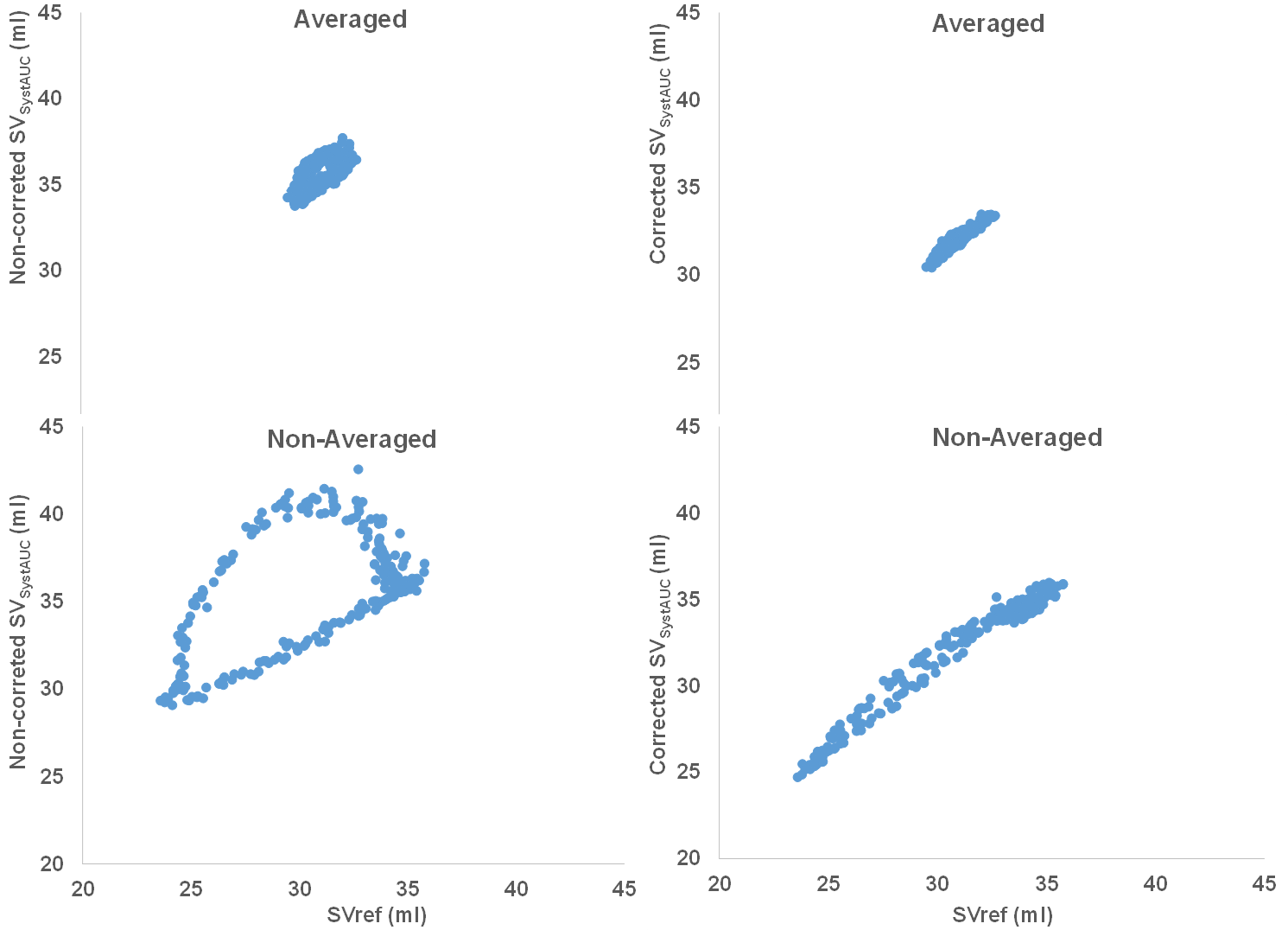


**eFigure 6.** Scatter plot of non-corrected (left) and corrected (right) SV_SystAUC_ with (top) and without smoothing by moving average (bottom) in one animal example.

A Bland-Altman analysis of averaged data was performed, and results are shown in eFigure 7 and eTable 1

**
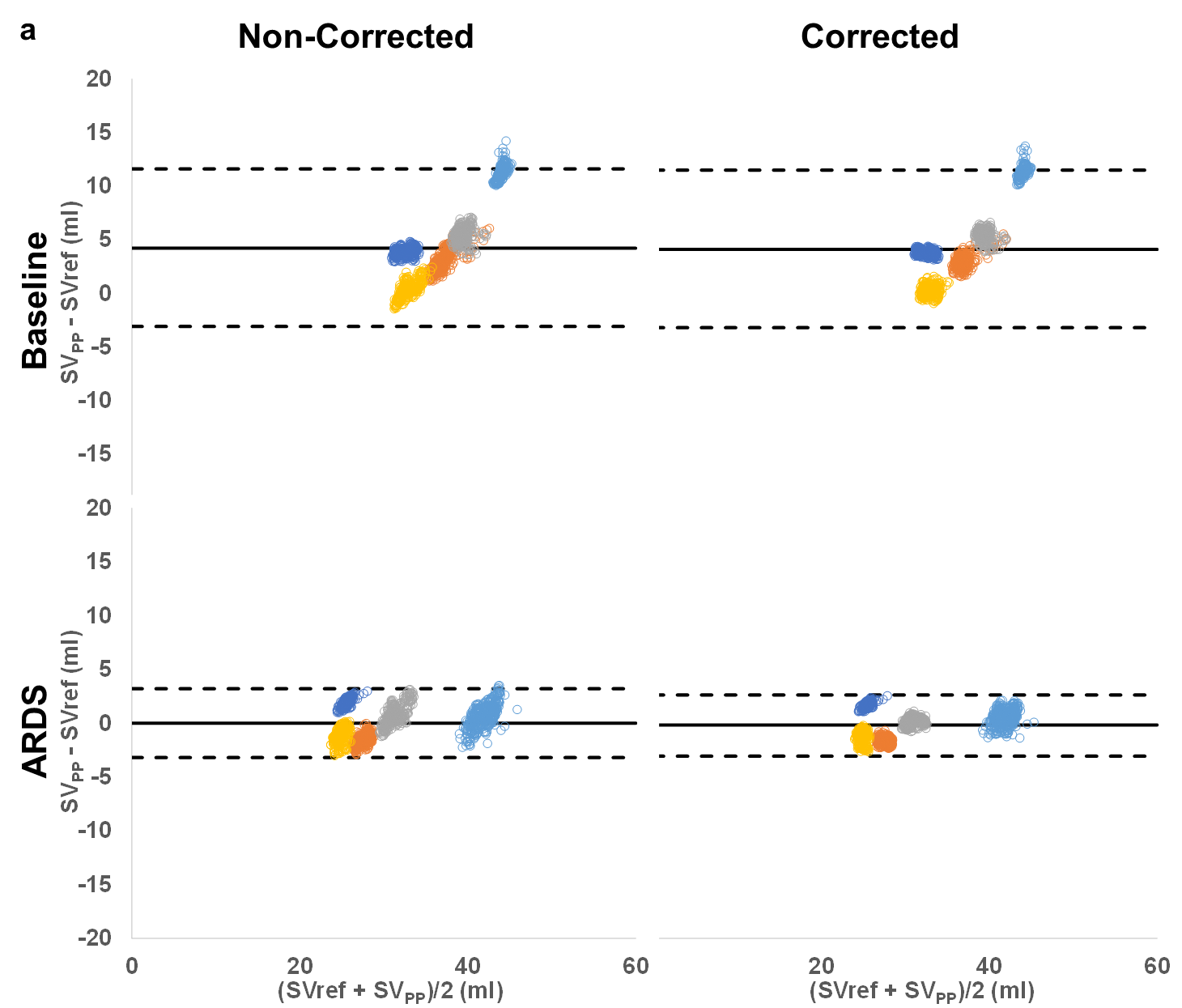
**

*
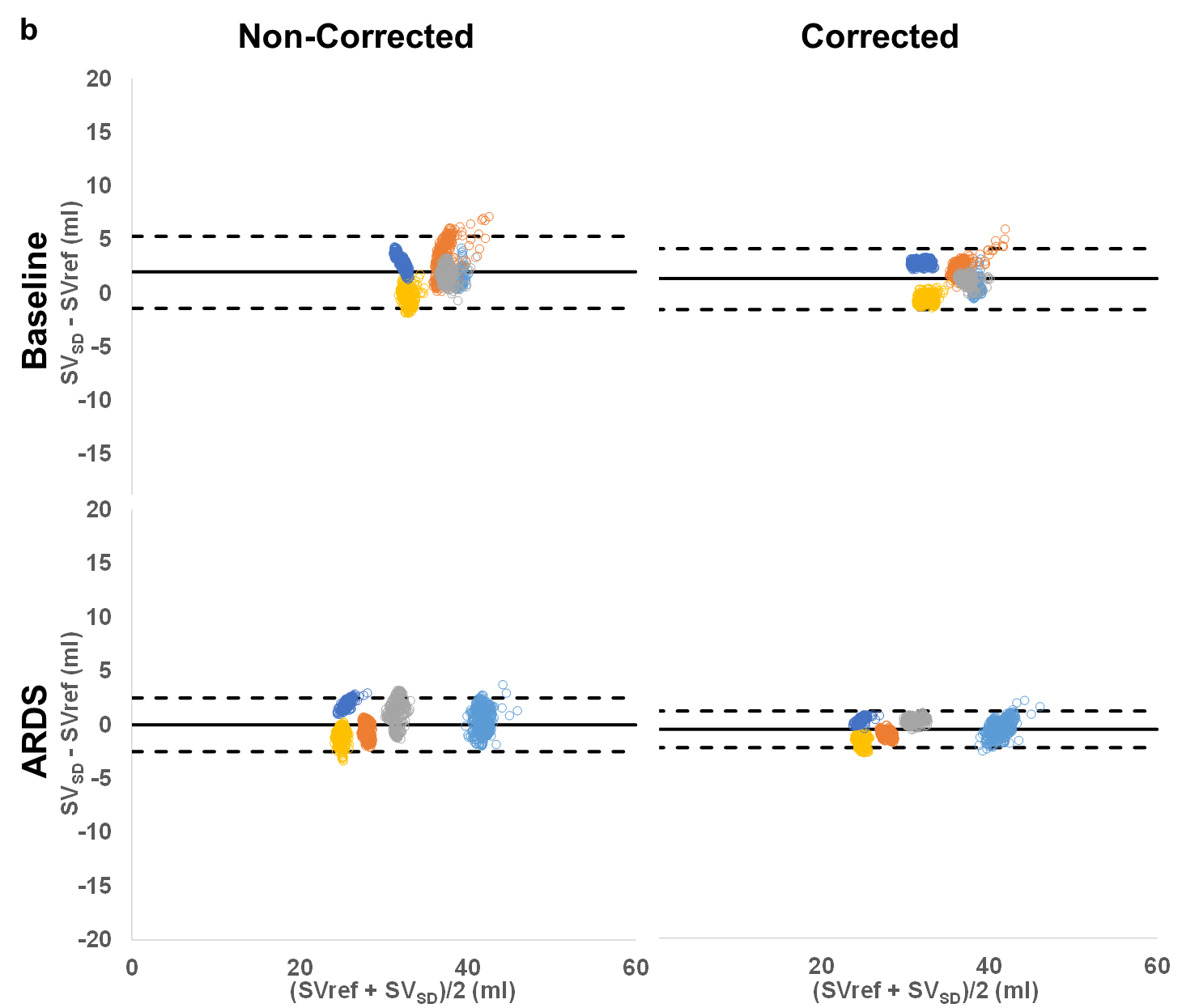
*

*
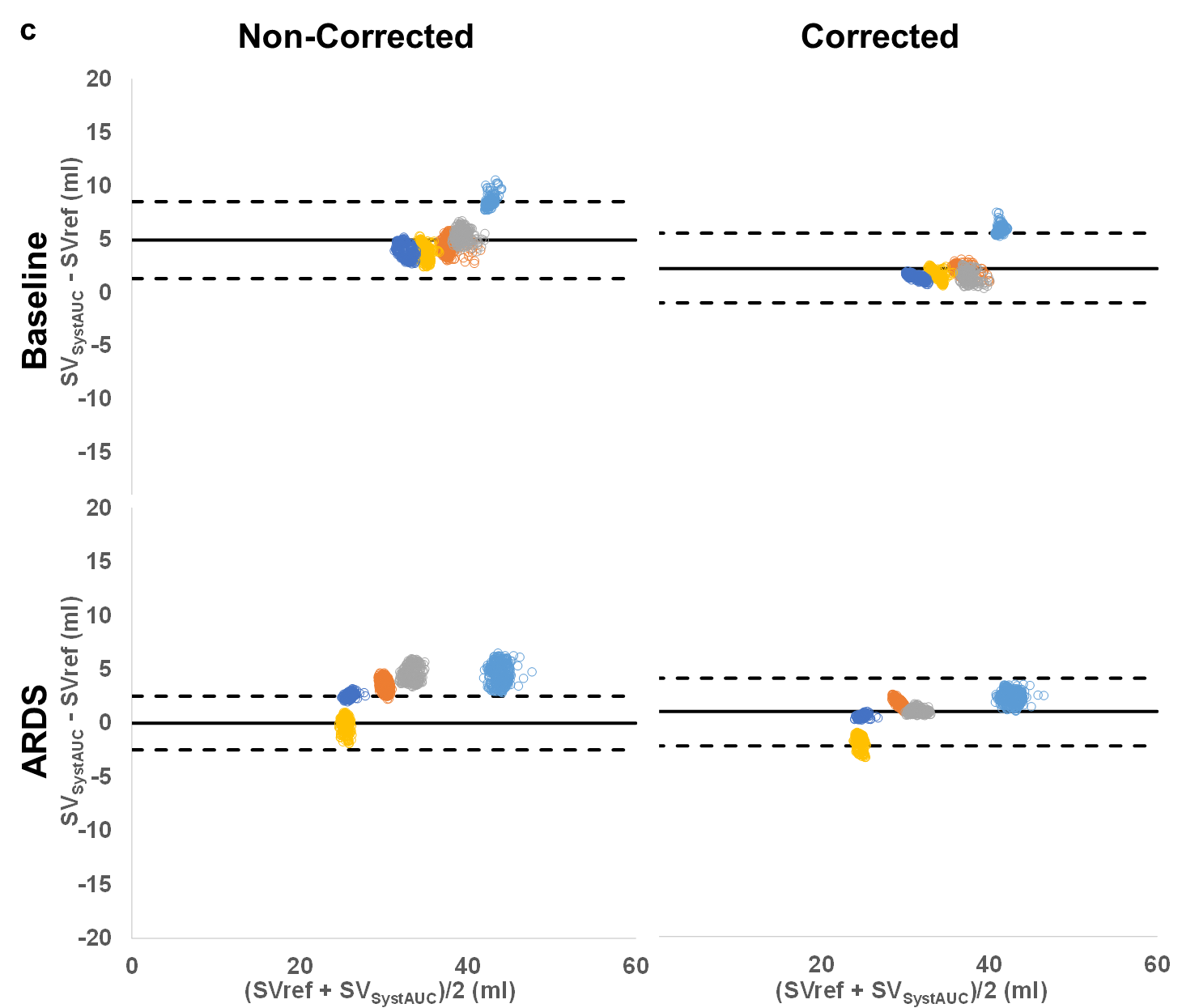
*

**efigure 7.** Bland-Altman plots calculated on averaged data. Limits of agreement were adjusted for repeated measures. Each colour represents an animal. **a.** SV obtained from PA Pulse pressure (SV_PP_). **b)** SV obtained from PA pressure standard deviation (SV_SD_). **c.** SV obtained from PA systolic area (SV_SystAUC_).

**eTable 1. Bland-Altman analysis of averaged stroke volume obtained from PA_PWA_ analysis and reference**

**Baseline**

Variable Correction Bias (ml) LoA(ml) Percentage error (%)

SV_PP_ Non-corrected 4.2 [-3.1 - 11.6] 19

Corrected 4.1 [-3.2 - 11.5] 19

SV_SD_ Non-corrected 2.0 [-1.4 - 5.3] 9

Corrected 1.3 [-1.6 - 4.1] 7

SV_SystAUC_ Non-corrected 4.9 [1.3 - 8.5] 9

Corrected 2.3 [-0.9 - 5.6] 8

**ARDS**

SV_PP_ Non-corrected 0.0 [-3.2 - 3.2] 10

Corrected -0.1 [-3.0 - 2.7] 8

SV_SD_ Non-corrected 0.0 [-2.5 - 2.5] 8

Corrected -0.5 [-2.2 - 1.2] 5

SV_SystAUC_ Non-corrected 3.1 [-0.8 - 7.0] 12

Corrected 1.0 [-2.2 - 4.1] 9

Stroke volume obtained from pulmonary artery pulse pressure (SV_PP_), standard deviation (SV_SD_) and systolic area (SV_SystAUC_) after applying a moving average with a 5 heartbeats window. LoA limits of agreement.

*Analysis of uncalibrated PA_PWA_ variables*

The effect of correction and lung conditions on the uncalibrated pulse wave analysis derived variable to estimate right ventricle stroke volume is shown in eTable 2. Only median and median absolute difference (MAD) are shown as the rest of evaluated parameter are the same as for the calibrated variables as they depend on the relative variability along the evaluated period.

*Phase shift analysis*

The results from the evaluation of the effect of phase shift in the correlation between the stroke volume calculated from the reference method and the estimated by the pulse wave analysis are shown in eTable 3.

**eTable 2. Uncalibrated pulse wave analysis derived variables used to estimate right ventricle stroke volume**

**Cond SVref (ml) PP (mmHg) SD (mmHg) SystAUC (mmHg/s)**

**Correction NC Co r NC Cor NC Cor**

**Median BL** 35.8 (2.9) 11.5 (4.6) 11.4 (4.4) 3.67 (1.51) 3.79 (1.59) 7.78 (1.26) 7.52 (1.26)^c^

**ARDS** 31.2 (7.1) 18.0 (2.0)^a^ 17.8 (2.2)^a^ 5.28 (0.76)^a^ 5.58 (0.90)^a^ 9.58 (1.99) 9.19 (1.91)^c^

**MAD BL** 2.2 (0.6) 1.1 (0.3) 0.5 (0.2)^c^ 0.15 (0.03) 0.20 (0.11) 0.38 (0.14) 0.24 (0.11)^c^

**ARDS** 2.1 (0.4) 1.7 (0.4) 0.8 (0.1)^c^ 0.23 (0.01)^a^ 0.28 (0.08)^a^ 0.50 (0.18) 0.38 (0.15)^c^

Median and MAD (median absolute difference) were obtained from all the beat-by-beat measurements during the studied period at each animal.

The representative value of each variable is the mean (SD) (according to distribution of data) for the 5 animals in the evaluated condition.

^c^ p < 0.05 for the correction ^a^ p < 0.05 for lung condition

**eTable 3. Evaluation of the phase shift between pulse wave analysis derived variables used to estimate right ventricle stroke volume and the reference method.**

**Cond PP SD SystAUC**

**Correction NC Co r NC Cor NC Cor**

**Phase shift (% of animals) BL** 20 20 80 20 100 0^c^

**ARDS** 60 0 100 0^c^ 100 0^c^

The value shown in the table corresponds with the percentage of studied pigs at each lung condition in which correlation between the stroke volume calculated by the reference method and by pulmonary artery pulse wave analysis improves in the cross correlation analysis in any lag between ±1 and ±4. ^c^ p < 0.05 for the correction
